# Multi-detector computed tomography and 3Tesla magnetic resonance imaging in assessment of COVID-19 intracranial complications

**DOI:** 10.1186/s43055-022-00767-3

**Published:** 2022-04-13

**Authors:** Ghada Sobhy Ibrahim, Buthaina M. Alkandari, Ahmed Mahmoud Elzeneini, Islam Ahmed Abo Shady, Ahmed Mohamed Housseini, Mohsen Ahmed Abdelmohsen

**Affiliations:** 1 Department of Radiodiagnosis, Faculty of Medicine, Tanta University, Algeish St, Tanta, 31527 Egypt; 2 FFR-RCSI, Dublin, Ireland; 3 Jaber Al Ahmad Hospital, Ministry of Health, Khalid Ben Abdulaziz Street, South Surra, Kuwait City, Kuwait; 4 Department of Radiodiagnosis, Nasser Institute for Research and Treatment, Kornish El Nile, Cairo, Egypt; 5 Department of Radiodiagnosis Faculty of Medicine, Mansora University, Mansora, Egypt; 6 Diagnostic Radiology Department, Faculty of Medicine, Suez Canal University, Ismailia, Egypt; 7 Department of Radio-Diagnosis and Intervention, Faculty of Medicine, University of Alexandria, Alexandria, Egypt; 8 Present Address: Faculty of Medicine, University of Alexandria, 10 Shamplion Street, Elazareeta, Alexandria, Egypt

**Keywords:** COVID-19, Neurological manifestations, Brain CT and MRI, Imaging findings

## Abstract

**Background:**

The novel worldwide coronavirus (COVID-19) pandemic, first appearing in Wuhan, China, has allured immense global attention. To our comprehension, this research work accommodates the largest isolation hospital-conducted cohort of coronavirus patients in which neuro-radiological complications were retrospectively assessed. To the present day, our full understanding of COVID-19 and its spectrum of diverse complications still remains insufficient. Moreover, the number of reported neurological complications albeit the global spread of the coronavirus pandemic is also widely lacking due to the constrained implementation of MR neuro-imaging in COVID-19 patients.

**Results:**

Forty-eight males and 26 females met the inclusion criteria, with a mean age 60.55 (ranged from 22 to 88 years old). The frequent clinical manifestation has impaired level of consciousness 55.4%. Most commonly recurring radiological findings were ischemic stroke 54.06% and parenchymal hematomas and hemorrhage 25.69%. Other less imaging brain findings were certain diagnostic entities, i.e., PRES, cerebral edema, leuko-encephalopathic WM abnormalities, microhemorrhages, vascular thrombosis and acute necrotizing encephalopathy. Soaring mortality rates correlated with serious neuro-radiological manifestations, being highest with infarction 57.5%, *p* = 0.908 and hemorrhage/hematomas 63.2%, *p* = 0.604.

**Conclusions:**

Intra-cranial complications were significantly detectable in COVID-19 infection and correlated with severity of illness. Outstanding higher mortality rates were associated with worsening neuro-radiological complications.

## Background

The novel worldwide coronavirus (COVID-19) pandemic, first appearing in Wuhan, China, has allured immense global attention [1]. The disease is caused by a member of the coronavirus family that affects humans and coronavirus 2 (SARS-CoV-2) and eventually leads to acute respiratory distress syndrome (ARDS) [2].

The exponential transmissibility of this novel outbreak of COVID-19 has provoked international healthcare systems around the globe, primarily due to the high risk of infectivity of the disease, even more paradoxically during its asymptomatic phase [3].

Symptoms of COVID-19 usually appear after a five-day incubation period. Although the greater number of patients suffered predominantly from fever, cough, dyspnea, fatigue and other upper respiratory tract infections, many other complications have been reported to affect other body systems, including gastro-intestinal, nephrological, cardiac and neurological systems, eventually leading to multi-organ failure [4].

While the majority of literature has widely focused on the typical respiratory manifestations of COVID-19, there has been a notable lacking in the thorough going documentation of neurological complications and their key radiological findings [5]. Neuro-invasion has been suggested through nasal infection along with the olfactory bulb [6]. Studies have generally agreed upon a 36–84% prevalence of neurologic symptoms among affected patients [7]. Disease neurotropism has been linked to a poor prognosis and severity [3].

There are plentiful emerging reports documenting a wide spectrum of associated neurological symptoms, ranging from milder presentations such as headache, dizziness, confusion, disorientation and depression, mounting up to much more serious complications such as stroke, hypoxic-ischemic brain insult, epilepsy, meningo-encephalitis, acute necrotizing encephalopathy, acute demyelinating and Guillain-Barré autoimmune disorders. The accumulating evidence for the variable detrimental neurological complications inflicted by the virus has promoted raise in awareness for the prompt radiological detection of such events [3].

### Aim of the work

The aim was to assess and describe the different neuro-radiological findings associated with COVID-19 infection.

## Methods

### Study participants

Adult patients with neurological symptoms who tested positive for real-time reverse transcription polymerase chain reaction (rRT-PCR) were included in this retrospective study. Those patients underwent advanced neuro-sectional imaging at our isolated center, Jaber Al-Ahmad hospital, from March 1, 2020, to November 8, 2020.

Inclusion criteria were: patients with positive nasopharyngeal swabs or PCR assays; severe COVID-19 in patients who required hospitalization with oxygen therapy; and patients with neurologic manifestations.

Exclusion criteria were: all patients with normal brain CT and MRI, lesions unrelated to the current event or patient refused to participate in the study, SARS-CoV-2 RT-PCR negative testing or none testing, out-patient or emergency department evaluation without hospital admission, pre-existing neurological manifestations, and missing or non-contributing CT or MR neuro-imaging data (lacking sequences and degrading artifacts). Exclusion criteria also included traumatic-related intra-cranial bleeds and other complications.

### Imaging criteria

Imaging was performed in accordance with the clinical routine. The scanner allocation was in keeping with the basis of clinical and logistical requirements.

### MRI studies

The study was done using a 3 Tesla MRI (Siemens Magnetom Skyra, Germany).

### MRI study preparation

No specific MRI preparations were done.

Infection control measures were taken (wearing masks and personal protective equipment).

#### MRI protocol

The sequences performed were.

- Sagittal T2 W (to plan all axials)
- 3D T1WI FSE
- Axial T2 W FSE
- Axial fluid attenuation inversion recovery sequence (FLAIR)
- Diffusion-weighted imaging (DWI), ADC (apparent diffusion coefficient)
- Susceptibility-weighted imaging (SWI).
- Coronal T2 W FSE
- MR arteriography and venography were done to some patients (TOF)
- The iodine-labeled and gadolinium-based contrast materials weren't utilized in our study.

### CT Studies

All CT brain studies were done using 128 and 256 rows (Siemens Edge Single source- Germany, Siemens Drive Dual Source-Germany respectively).

### Specific CT study preparation

No specific CT preparations were done.

Infection control measures were taken (wearing masks and personal protective equipment).

### The CT protocol

Non-contrast cross-sectional imaging was performed. Sub-millimeter high spatial resolution imaging was acquired with multi-planar reconstructed imaging in three planes at a 5-mm slice thickness. MSCT brain was without IV contrast with MPR (sagittal and coronal images).

### Visual assessments and imaging interpretation

Anonymous cross-sectional studies were presented to the involved neuro-readers on standard picture archiving and communication systems (General Electric, Germany). A review of cross-sectional studies was performed by three experienced neuro-radiologists (I.S., G.S. and A.Z; with 12, 11 and 10 years of experience in neuro-radiology, respectively). Reviewing neuro-radiologists were blinded to all patient data.

### Ethics approval and consent to participate

Approval for this study was obtained from the Research Ethics Committee of our medical institute. All study procedures were carried out in accordance with the Declaration of Helsinki regarding research involving human subjects. Written consent was waived.

## Results

### Patient demographics

This retrospective study overall totaled 74 consecutive patients admitted to our hospital during the period from March 1, 2020, to November 8, 2020, with confirmed SARS-CoV-2 infection and neurologic manifestations. Patient demographics ranged from 22 to 88 years old, with a mean age of 60.55, of whom 48 (64.86%) were males and 26 (35.14%) were females. They had all undergone cross-sectional neuro-imaging on the basis of routine clinical indications at Jaber Al-Ahmad Hospital.

The hospitalized patients during the time frame of the study were evaluated by the hospital neurology team. A total of 114 patients suffered from neurological manifestations. Testing positive for SARS-CoV-2 RT-PCR, 74 patients (64.9%) of the patients included in the analysis presented with de-novo neurological dysfunction and COVID-19-related neurological findings and complications. On the other hand, 40 patients (35.1%) of the patients during the same time frame of the study testing positive for SARS-CoV-2 were admitted and hospitalized with normal or unrelated COVID-19 neurological findings.

### Risk factors, clinical and laboratory findings

Risk factors of the studied patients are as follows (Table 1): Smoker patients represented 34 patients (45.95%), obesity was noted in 12 patients (16.22%), and ARDS in 60 patients (81.08%). The most common underlying systemic co-morbidities were hypertension in 50 patients (67.57%), diabetes mellitus in 7 patients (9.46%), dyslipidemia in 5 patients (6.8%) and ischemic heart disease (with hypertension) in 4 patients (5.4%). Patients who underwent CABG were 8 patients (10.8%) and Crohn’s disease in 2 patients (2.7%).

**Table 1:** Clinical characteristics of the studied patients

	Number of the patients (n = 74)	Percentage %
*Smoking history*		
Smoker	34	45.95
X-smoker	9	12.16
Never smoker	24	32.43
Unknown	7	9.46
*Body Mass Index (range kg/m*^*2*^*)*		
Normal: 18.5–25	29	39.19
Overweight: 25–30	33	44.59
Obese: over 30	12	16.22
Lung Disease		
Asthma	3	4.05
COPD	2	2.7
Chronic interstitial lung disease	2	2.7
ARDS	60	81.08
Lung metastasis	1	1.35
None	6	8.11
*Medical history*		
Hypertension		
Alone	8	10.8
With DM	20	27.02
With DM and CABG	8	10.8
With IHD	4	5.4
With chronic renal disease	6	8.11
With liver impairment	1	1.35
With CRD and liver impairment	3	4.05
DM		
Alone	2	2.7
With dyslipidemia	5	6.8
Crohn's disease	2	2.7
Normal	15	20.27

Much more detrimental previous neurological illness was represented by 17 patients (22.97%) as: stroke 8 (10.8%), epilepsy 2 (2.7%), Parkinsonism 4(5.4%), Alzheimer's disease 2 (2.7%) and cerebral palsy 1 (1.35%).

The duration between neurological signs/symptoms and cross-sectional imaging ranged from 1 to 4 days, with a mean interval of 2 days in 41 patients (55.4%).

The presented neurological manifestations were classified into **mild** manifestations, which were found in 47 patients (63.5%) at the time of admission, such as headache, myalgia and anosmia, and **severe** manifestations in the form of impaired conscious level were 41/74 patients (55.4%), with all included patients with imaging findings of acute or subacute infarct, and 40/74 (54.06%) had focal neurological deficits on clinical examination (as left or right-sided weakness). Similarly, patients with parenchymal hematoma or/and hemorrhage 19/74 (25.69%) had either focal neurologic deficits or a history of syncopal attack.

### The prevalence of neuro-imaging findings

Fifty-eight patients had only CT (78.4%), 9 patients had only MRI (12.2%), (2 examinations were MRA with contrast), and 7 patients underwent both CT and MRI (9.5%).

**Fig. 1:**
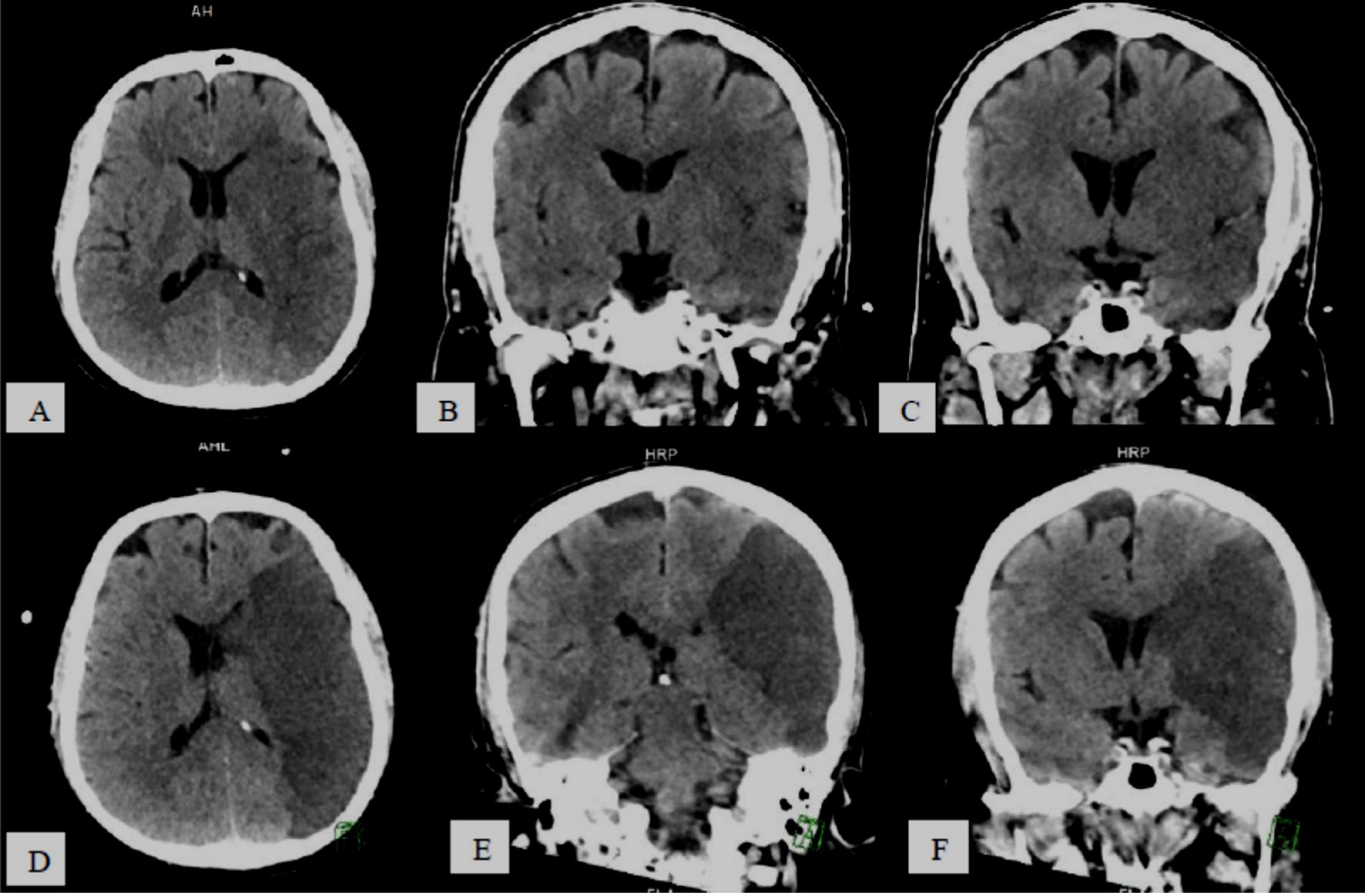
58-year-old COVID-19 male patient with right-sided weakness and impaired level of consciousness, **A–C** serial axial and coronal reformatting CT brain study without contrast, showing large ill-defined left fronto-temporo-parietal cortical and subcortical hypodense area with mild mass effect in form of effacement of cortical sulci and ispilateral lateral ventricle. **D–F** serial axial and coronal reformatting CT brain study follow-up after 1 day shows massive progressed left fronto-temporo-parietal *acute non-hemorrhagic infarction* (left MCA territory) with increase mass effects

**Fig. 2:**
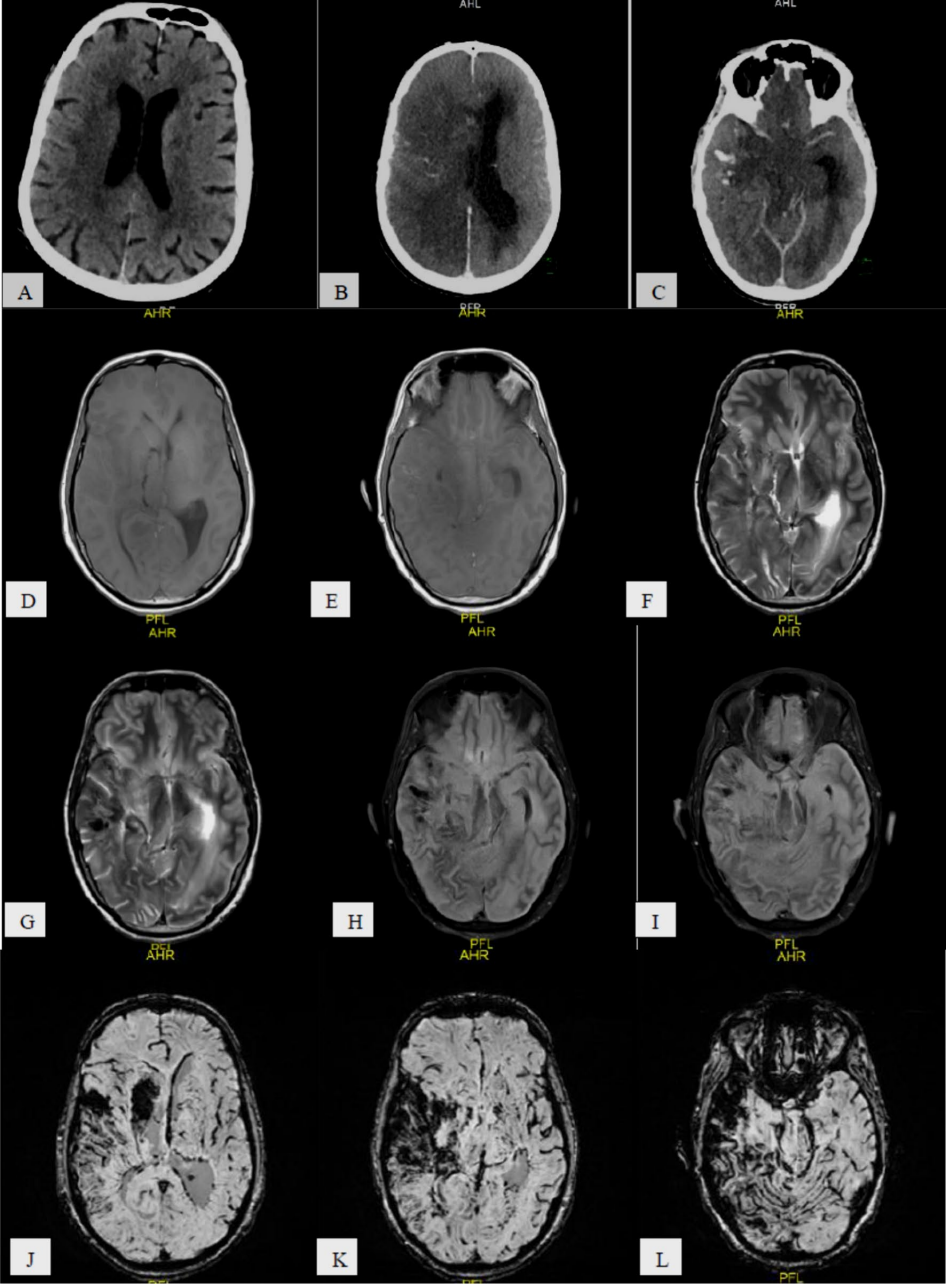

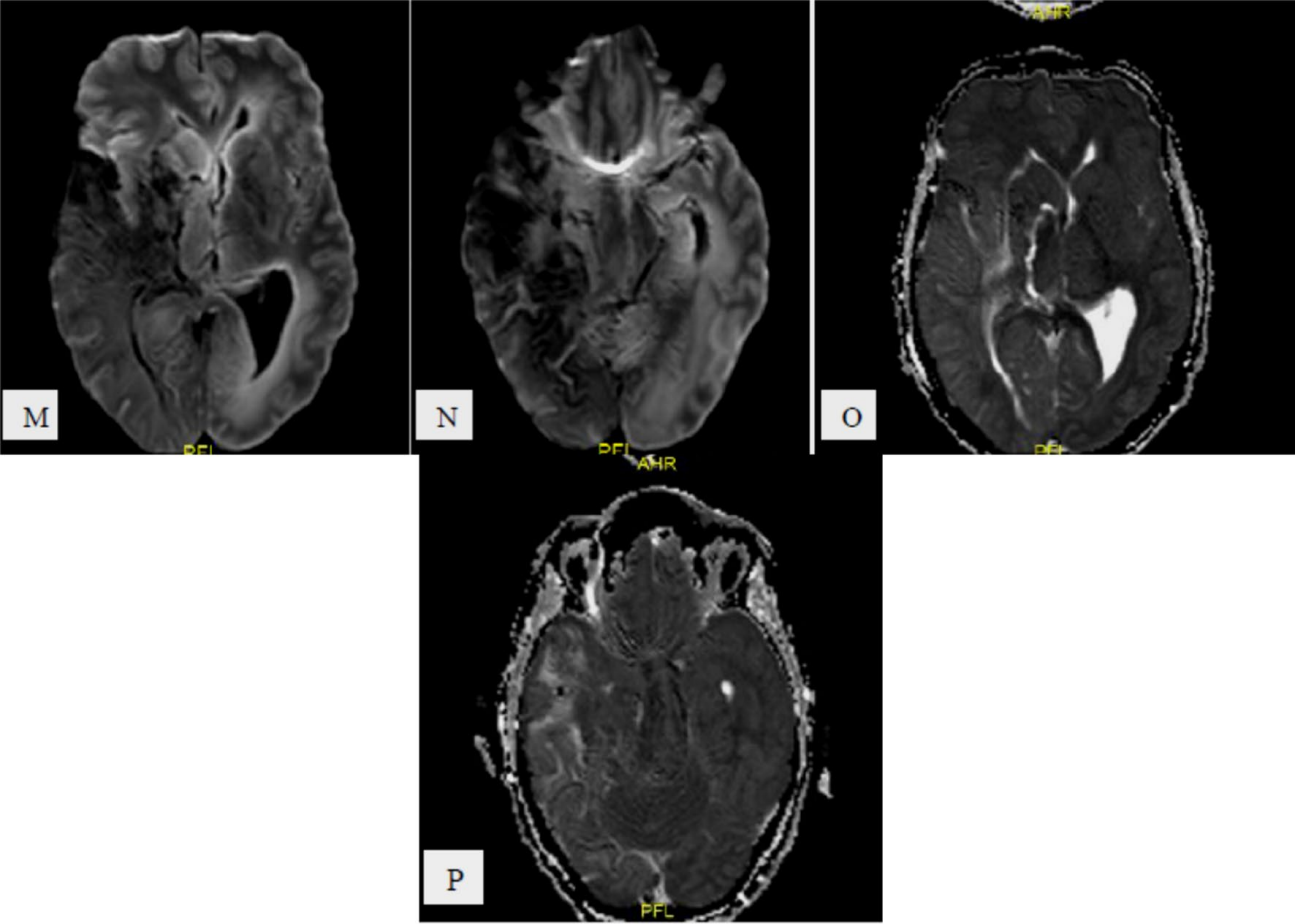
76-year-old COVID-19 male patient with sudden onset of left sided weakness (**A**) axial CT image shows right temporo-parital ill-defined deep white matter periventricular hypodensity, **B**, **C** Follow-up 2 days later of serial axial CT images shows progressive course regarding the size of the right fronto-temporo-parieto-occipital large right cerebral infarction with hemorrhagic component with sub-falcine herniation. **D**, **E** T1 WI, **F**, **G** T2 WI, **H**, **I** FLAIR, **J–L** SWI, **M**, **N** DWI, **O**, **P** ADC map**,** 7 days later MR follow up shows *aging* *right* *fronto-temporo-parieto-occipital* *hemorrhagic* *infarction*

**Fig. 3:**
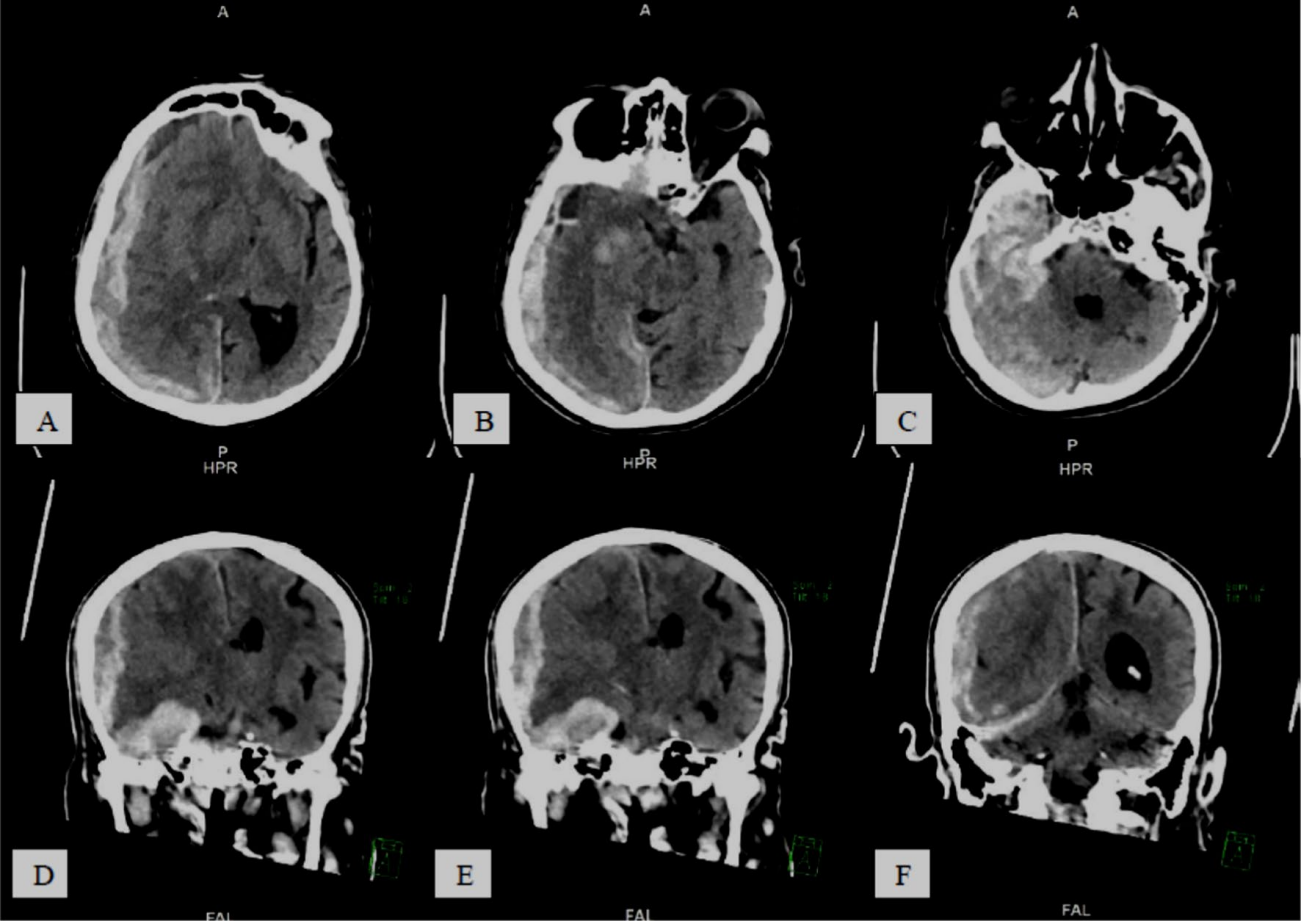
88-year-old COVID-19 male patient with sudden onset of left sided weakness and syncopal attack, under umbrella of anticoagulant, **A–C** and **D–F** serial axial and coronal CT images, respectively, show right fronto-temporo-parital extra-axial acute on top of chronic subdural hematoma with mid line shift, sub-falcine and uncal herniations

**Fig. 4:**
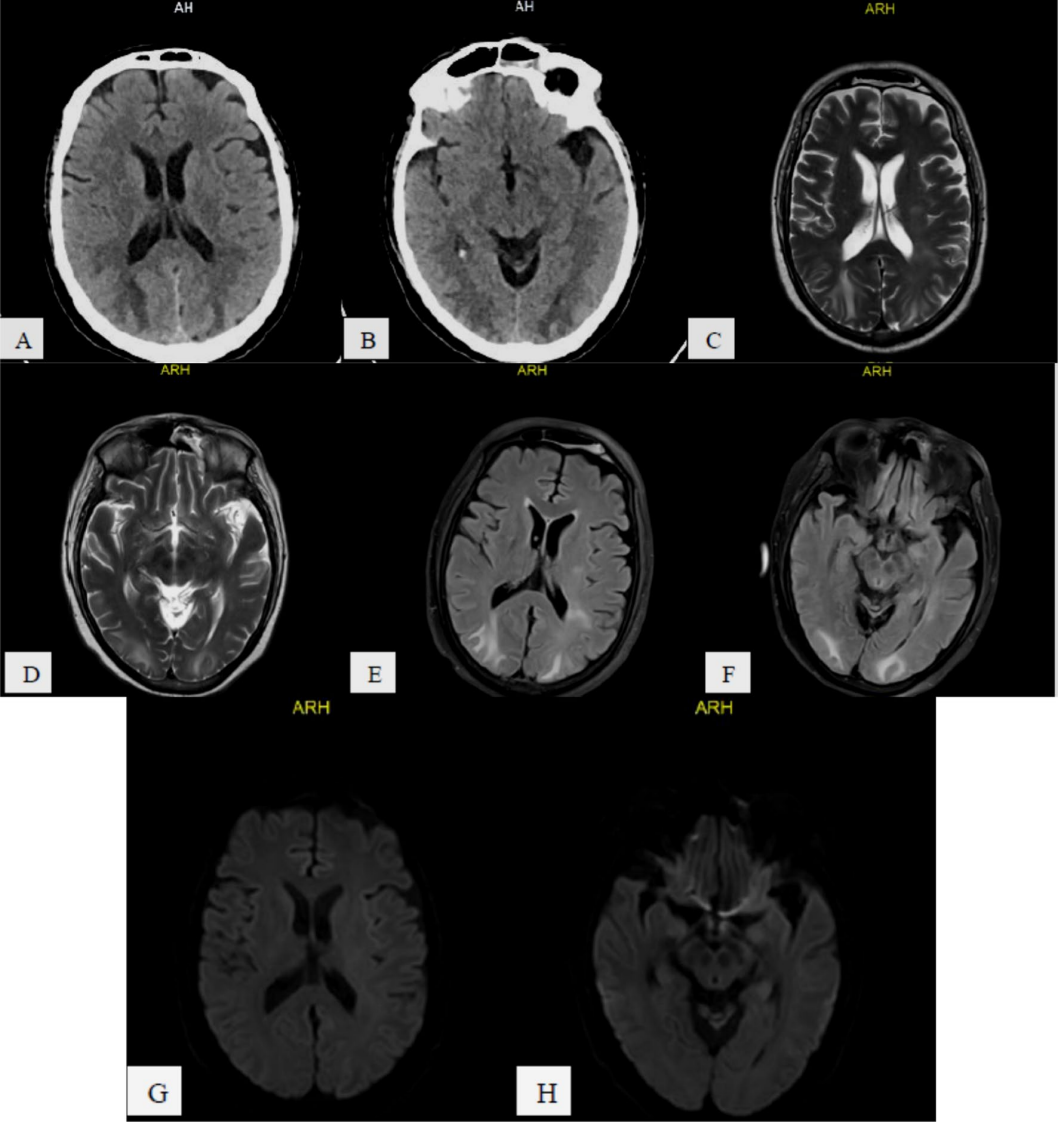
47-year-old COVID-19 male patient with first time GTC seizure after prolonged ICU admission **A**, **B** axial CT images, **C**, **D** T2 WI, **E**, **F** FLAIR, **G**, **H** DWI showing bilateral posterior parieto-occipital and to less extent high frontal subcortical and deep white matter hyperintense signal noted on T2 and FLAIR images with morphological vasogenic edema pattern. No significant mass effect. It shows free diffusion features are impressive of morphological *PRES* (*posterior* *reversible* *encephalopathy* *syndrome*)

**Fig. 5:**
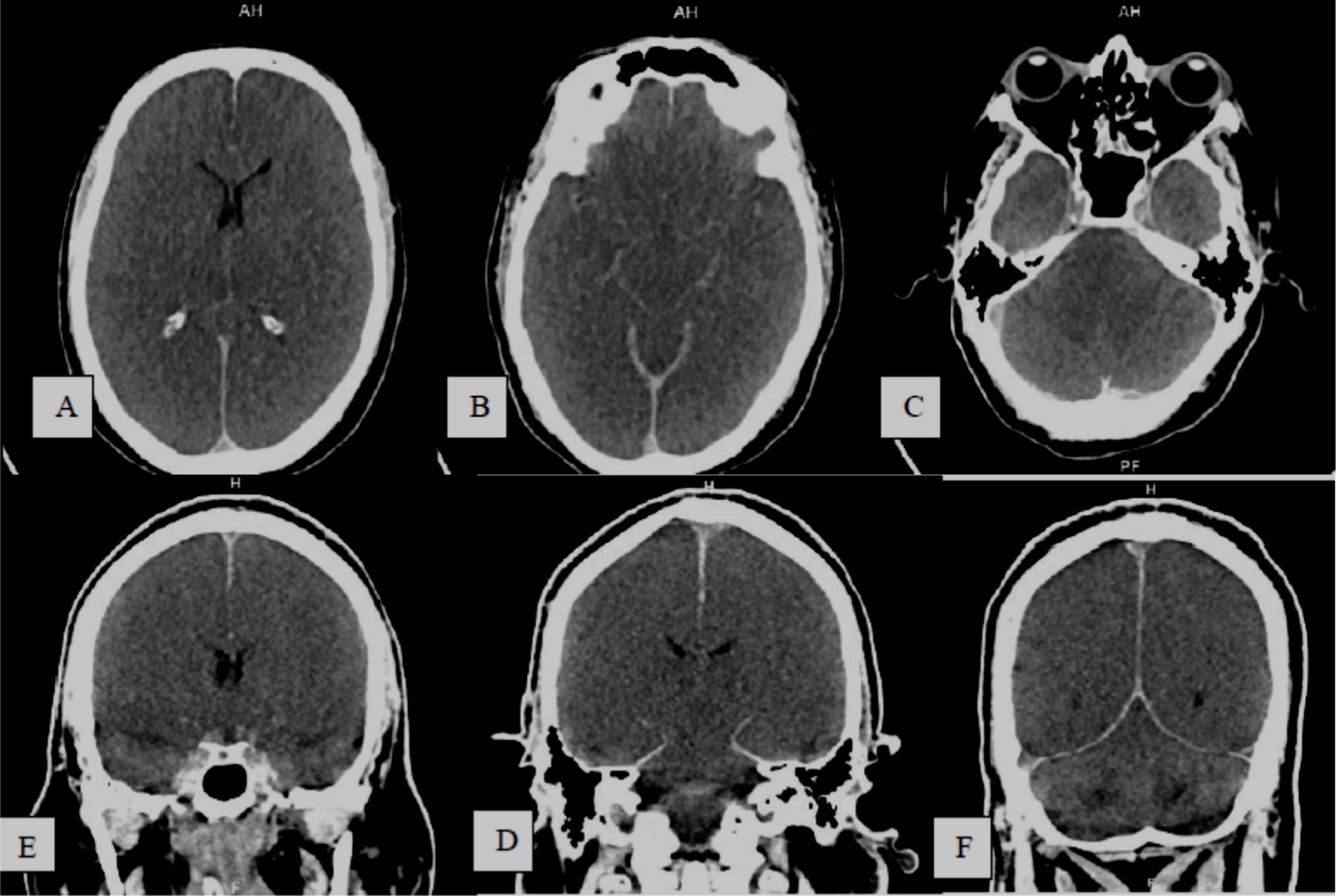
60-year-old COVID-19 male patient with impaired level of consciousness, **A**–**C** serial axial CT brain study without contrast, **D**–**F** serial coronal reformatting images, showing poor gray/white matter differentiation with diffuse brain parenchyma hypodensity reflecting *diffuse* *cerebral* *edema*

**Fig. 6:**
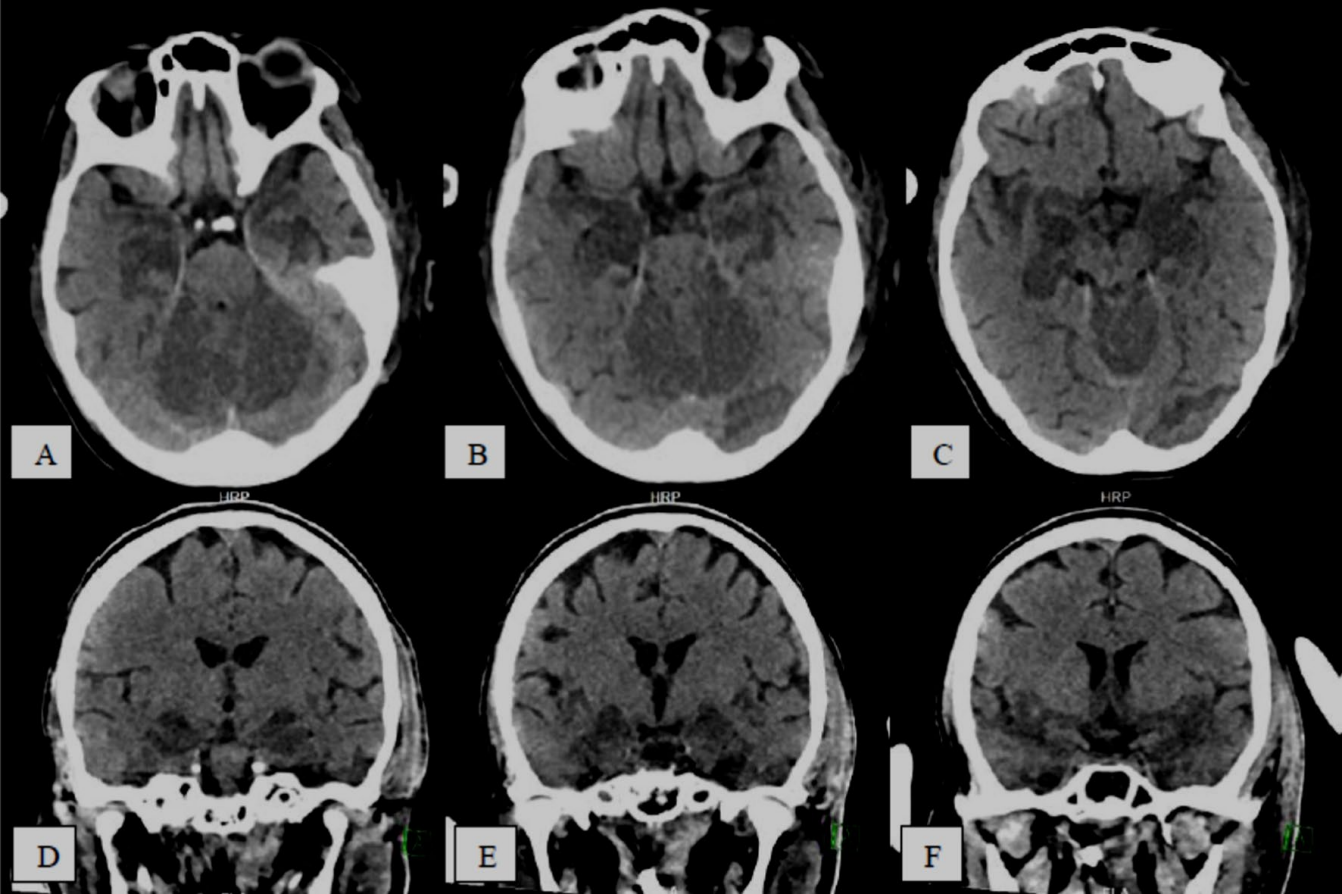
22-year-old-female COVID-19 positive patient complaining of worsening pneumonia and difficult to oxygenate. Presented with impaired level of consciousness, **A**–**C** and **D**–**F** serial axial and coronal CT images, respectively, show bilateral cerebellar and medial temporal lobe hypo-attenuation with relative loss of gray white matter differentiation, suggestive of *hypoxic* *ischemic* *encephalopathy* *(global* *hypoxic-ischemic* *injury)* *with* *COVID-19* *related* *leukoencephalopathy* *changes*

**Fig. 7:**
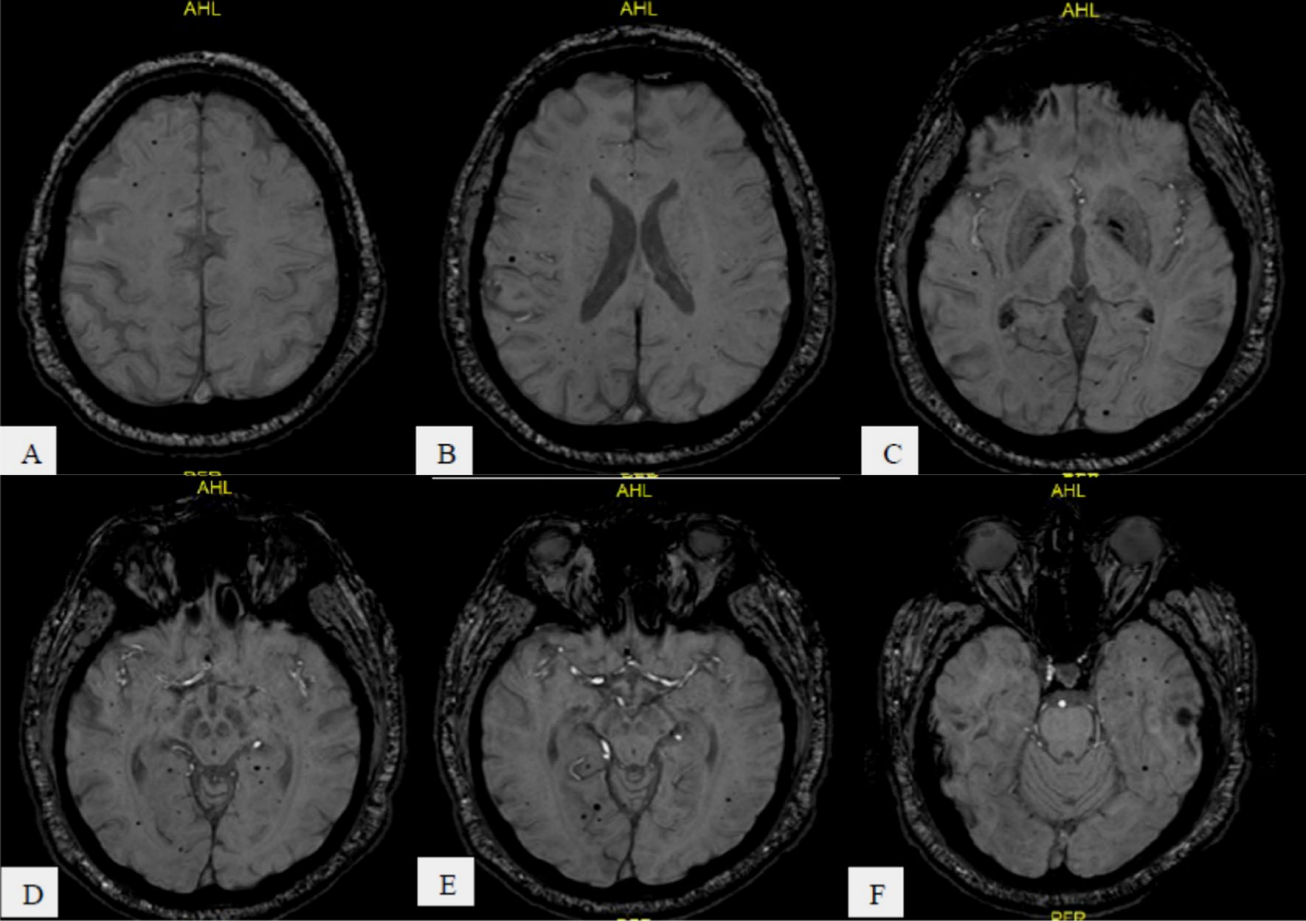
42-year-old COVID-19 male patient with impaired level of consciousness, **A**–**F** serial axial MRI SWI sequences show innumerable variable size multiple scattered minute discrete foci of abnormal blooming signal that seen in both cerebral deep white matter on suggesting of COVID-19-related microhemorrhage

**Fig. 8:**
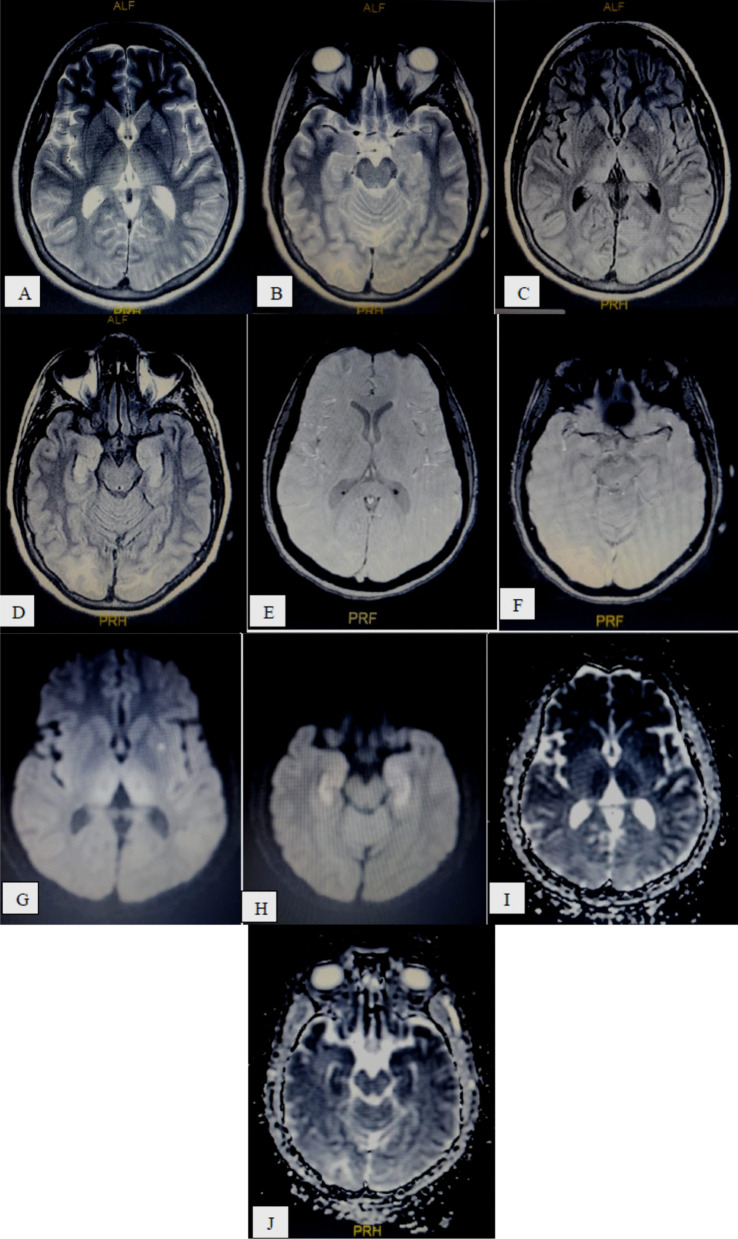
51-year-old-female COVID-19 positive patient presented with impaired level of consciousness, **A**, **B** T2 WI, **C**, **D** FLAIR, **E**, **F** SWI, **G**, **H** DWI and **I**, **J** ADC map showing bilateral thalamic, bilateral occipital subcortical, bilateral temporal cortical and subcortical T2 and FLAIR hyperintense SI with restricted diffusion (hyperintense in DWI and hypointense in ADC map) represented acute necrotizing encephalopathy without hemorrhage, with left putamen acute infarction

The most common presented CT and/or MR specific neuro-radiologic diagnoses were: *stroke* 40/74 patients (54.06%), subdivided into: non-hemorrhagic 28/40 patients (70%) (Fig. 1) and hemorrhagic 12/40 patients (30%) (Fig. 2),* hemorrhage and hematomas* 19/74 patients (25.69%), subdivided into: *extra-axial* 11/19 (57.9%) including subdural 7/11 (63.64%) (Fig. 3) and SAH 4/11 (36.36%), *intra-axial* 7/19 (36.84%) including cerebral 5/7 (71.42%), brain stem 1/7 (14.29%) and cerebellar 1/7, (14.29%), and *both intra/extra-axial* 1/19 (5.26%), *PRES* 3/74 patients (4.05%) (Fig. 4),* diffuse brain edema* 2/74 patients (2.7%) (Fig. 5), *WM COVID-related leuko-encephalopathy* 3/74 patients (4.05%) (Fig. 6), *microbleeds* 3/74 patients (4.05%) (Fig. 7), *vascular pathology* 2/74 patients (2.7%), one had left transverse sinus thrombosis and another had thrombosis of extra-cranial portion of left internal carotid artery, and *acute necrotizing encephalopathy* 2/74 patients (2.7%) (Fig. 8).

All the above-mentioned frequent neuro-imaging findings are summarized and tabulated in Table 2.

**Table 2:** Classification of neurological findings

Findings	Neurological Findings (n = 74)					
	N	%	Sub-types	N	%	
Stroke	40/74	54.06	Non hemorrhagic	28/40	70	
			Hemorrhagic	12/40	30	
Parenchymal hematoma or/and hemorrhage	19/74	25.69	Extra-axial 11/19 (57.9%)	Subdural	7/11	63.64
				SAH	4/11	36.36
			Intra-axial 7/19 (36.84%)	Cerebral	5/7	71.42
			Both 1/19 (5.26%)	Brain Stem	1/7	14.29
				Cerebellar	1/7	14.29
PRES	3/74	4.05				
Cerebral edema	2/74	2.7				
Leuko-encephalopathic WM abnormalities	3/74	4.05				
Microhemorrhage	3/74	4.05				
Vascular thrombosis	2/74	2.7				
Acute necrotizing encephalitis	2/74	2.7				

There were 33 patients (44.6%) under the umbrella of anti-coagulant therapy. 14 patients (73.7%) developed hemorrhage and hematomas. The admission period of the studied patients ranged from 4 to 75 days; the mean range of admission was 11–20 days in 24 patients (32.43%). Patients' outcomes were categorized as follows: 18 patients (24.32%) were discharged, 13 (17.57%) remained hospitalized, and 43 (58.11%) deceased. The epidemiologic profile and clinical characteristics of dead and alive patients are detailed in Table 3.

**Table 3:** Epidemiologic profile and clinical characteristics

Demographic and clinical parameters	All patients (n = 74)	Died patients (n = 43)	Alive patients (n = 31)	*p*-value
Sex (male/female)	48/26	27/16	21/10	0.66
Age (years), mean/range	61/23–88	64/23–88	48/45–78	0.001*
Time from onset of symptoms* to brain imaging (days) mean/range	2/1–4	2/1–4	2/1–4	0.58
Clinical characteristics				
History of smoking	34	23 (67.6%)	11 (32.3%)	0.125
Obesity	12	10 (83.3%)	2 (16.7%)	0.053
ARDS	60	29 (48.3%)	31 (51.7%)	0.0004*
Medical history	59	40 (93.02%)	19 (61.3%)	0.0008*
Previous neurological illness	17	8 (47.1%)	9 (52.9%)	0.292
Follow-up by CT after 2 days	9	4 (44.4%)	5 (55.6%)	0.375
Clinical manifestation				
Impaired consciousness	22	11 (50%)	11 (50%)	0.357
Neurological findings				
Infarction	40	23 (57.5%)	17 (42.5%)	0.908
Hemorrhage and hematomas	19	12 (63.2%)	7 (36.8%)	0.604
PRES	3	0 (0.0)	3 (100%)	0.037*
Cerebral edema	2	1 (50%)	1 (50%)	0.813
WM leucoencephalopathy-related COVID-19	3	2 (66.7%)	1 (33.3%)	0.759
Microhemorrhage	3	3 (100%)	0 (0.0)	0.259
Vascular thrombosis	2	0 (0.0)	2 (100%)	0.172
Acute necrotizing encephalitis	2	2 (100%)	0 (0.0)	0.506
Admission period				
11–20 days	24	16 (66.7%)	8 (33.3%)	0.301

Used tests: Mann–Whitney U test, Chi-square and Fisher exact test *statistically significant (if *p* < 0.05)

The most common abnormal clinical laboratory findings of dead and alive patients, including white blood cell count, lymphocyte count, platelet count, C-reactive protein and D-dimers, are detailed in Table 4.

**Table 4:** Laboratory findings

Laboratory findings	Normal range	All patients (n=74)	Died patients (n=43)	Alive patients (n=31)	p values
White blood cell count and range, × 109/L (N=41)	(4–10)	13.3 (10.0–13.7)	13.6 (10.8–13.7)	13.4 (10–12.2)	0.20
Lymphocyte count, range × 109/L (N=36)	(1.5–4)	1.09 (0.52–1.4)	1.05 (0.77–1.4)	1.11 (0.52–1.4)	0.17
Platelet count, × 109/L (N=38)	(150–450)	297 (144–340)	282 (202–340)	290 (144–323)	0.38
C-reactive protein and range mg/L (N=53)	< 4	57.5 (16–140)	42 (22–137)	71 (16–140)	0.001*
D-dimers and range mg/L (N=44)	< 0.5	2.7(0.7–3.8)	3.2 (2.3–3.8)	1.6 (0.7–3.3)	0.001*

Used tests: Mann–Whitney U test, parameters described as median (range) *statistically significant (if *p* < 0.05)

*N* is the total number of patients with available data, and n the number of positive patients.

### Statistical analysis and data interpretation

Data were fed into the computer and analyzed with IBM SPSS Corp., which was released in 2013. IBM SPSS Statistics for Windows, Version 22.0. Armonk, NY: IBM Corp. Qualitative data were described using number and percent. Quantitative data were described using median (minimum and maximum) for nonparametric data after testing normality using Kolmogorov–Smirnov test. Mann–Whitney *U* test was used to compare two independent groups of nonparametric variables. Chi-square test and Fisher exact test were used for categorical variables.

## Discussion

We hereby present our experience stemmed from this current retrospective observational study, which has been conducted at Jaber Al-Ahmad Hospital. Neuro-radiologic observations were analyzed and reported in a cohort of patients, consecutively hospitalized at our institute, with positive coronavirus 2019 disease.

In this study, we had reviewed the intracranial neuro-radiological findings in patients systemically infected with COVID-19; this is the best of our knowledge and systematic review describing neuroimaging results in patients with COVID-19 infection.

In our study, we observed that COVID-19 male patients had more neurological complications 48/74 (64.86%); our result were near in line with Chougar et al. [8]. Males were more affected 48/73 (65.8%).

At our institution, COVID-19 ill elderly patients above 60 years old with various risk factors were more vulnerable to cerebro-vascular disease were 42 patients 56.8% this result agreed with Sanaz Katala et al. [1].

Many studies had suggested that coronaviruses have neuro-invasive properties, although in the absence of respiratory symptoms. During COVID-19 outbreak, several new reported cases suggested the association between coronavirus and neurological symptoms. Our study found those mild symptoms such as headache; myalgia and anosmia were among the most mutual neurological manifestations associated with SARS-CoV-2 infection, Munhoz et al., [9]. study supported our observations.

The most frequent neurological manifestations in included patients were impaired consciousness not explained by therapy 41/74 (55.4%); Chougar et al. [8] studied impaired consciousness representing 39/73 (53.4%).

The majority of the included patients with imaging findings of acute/subacute infarct had focal neurological deficits on clinical examination. Similarly, patients with acute hemorrhage had either focal neurologic deficits or a history of syncopal attack; this observation was as Mao et al. [10].

Acute respiratory distress syndrome and hypoxemia that can be seen in COVID-19 patients can affect mental status. While alteration in mental status warrants clinical neurological examination, failure of clinical examination to detect a focal neurologic deficit or no history of syncope or fall, the neurological imaging may not be particularly revealing, an observation that is concordant with prior studies of brain imaging in acute altered mental status [11].

Many studies believed that hyperimmune response due to cytokine storms may explain these neurologic manifestations, while the others had proposed direct invasion by virus of human brain cells by hematogenous, transcribrial or neuronal dissemination retrograde routs. In addition, the neurotropism of SARS-CoV-2 might mediate by angiotensin-converting enzyme 2 receptors (ACE2) that were expressed by brain capillary endothelial cells. Rupture of cerebral endothelium leads to irreversible brain damage that contributed in pathophysiology of SARS-CoV-2 neurologic manifestations [12].

Diversified neurological clinical manifestations were described in COVID-19 patients. The data were different on this study. Available associated neurological findings were infarction, hemorrhage (in different sites), PRES, cerebral edema, leuko-encephalopathic WM abnormalities, microhemorrhage, vascular thrombosis and acute necrotizing encephalopathy.

Neuro-imaging modalities such as CT and MRI had revealed many findings in the context of different clinical scenarios. 35.1% of our patients during the same time frame of the study testing positive for SARS-CoV-2 were admitted and hospitalized with normal or unrelated COVID-19 neurological findings, while in Sanaz Katala et al. [1] study they found 40% of their COVID-19 infectious patients had displayed normal results.

While our other COVID-19 patients had demonstrated imaging abnormalities in different areas of the brain, including ischemic stroke 54.06%, hemorrhage 25.69% and other neurological abnormalities, Sanaz Katala et al. [1] found that the most common neuroradiologic abnormality were both ischemic and hemorrhagic cerebrovascular events were seen among 27% COVID-19 patients. Other many studies were found the ischemic and hemorrhagic cerebro-vascular manifestation lower percentage than our results as in Munhoz et al*.* [9] found 2.8–5.7%, Mao [10] and Asadi-Pooya et al. [6] both reported that ischemic or hemorrhagic CVD account for 5–5.7% of neurologic manifestations associated with COVID-19.

The stroke pathogenesis in COVID-19 may be from the impairment of coagulation or endothelial functions. Coagulopathy may be related to the thrombophilic effects of systemic inflammation; the presence of lupus anti-coagulant and anti-phospholipid antibodies has also been reported. [13–15].

Although high incidence of deep vein thrombosis and elevated D-dimer levels in COVID-19 patients was reported, Tang et al., [16] did not find evidence of cerebral venous thrombosis in their cohort; we disagreed with their observation as we found two patients with vascular thrombosis, one left transverse sinus thrombosis and another had left internal carotid artery thrombosis.

Parenchymal hematomas and hemorrhage were found in 19 (25.69%) patients, most (n = 14) of whom had hemorrhages attributed to anticoagulation, a finding that highlights the risks of initiating anticoagulant therapy in response to the prothrombotic features of patients with COVID-19.

Microhemorrhages were found in 3 patients (4.05%), demonstrating innumerable variable size multiple scattered minute discrete foci of abnormal blooming signal that seen in both cerebral deep white matter without involvement of corpus callosum; our results were not in keeping with another recent observational study describing 4 patients with COVID-19 with microhemorrhage in the corpus callosum [17].

Blood spread to the CNS is proposed to be due to viral related to ACE2 receptors in the endothelial capillary, resulting in damaging to its lining. This could lead to increase the blood–brain barrier permeability and loss of hemostatic regulation, leading to cerebral edema [18].

In our result, this mechanism could explain PRES cases, as has been recently seen in 2 other cases of PRES with COVID-19 (19). However, each of our 3 patients with PRES also had the typical risk factors of chronic renal disease and elevated blood pressure.

Three of included patients (4.05%) had microhemorrhage; Lin et al. [18], 3/51 (5.8%) had patients with a microhemorrhage pattern compatible with critical illness-associated microbleeds.

In our results, three patients (4.05%) had only leuko-encepalopathic WM abnormalities, and also three patients (4.05%) had only microhemorrhage and we did not find combination of these both findings in our study. Radmanesh et al. found that four patients (14.8%) had only leukoencephalopathy, one patient (3.7%) had only microhemorrhages, and six patients had a combination of both [17].

In our study the leuko-encephalopathic WM abnormalities were presenting as abnormal high signal intensities on T2WI and FLAIR images were predominantly depicted at the periventricular and deep subcortical white matter. In contrary, Radmanesh et al. showed predilection for the corpus callosum and juxta-cortical WM [17].

We also found that two of our patients with COVID-19 had evidence of cerebral edema, similar to those shown in a recent case report of a patient with fulminant cerebral edema [19, 20].

In our study we found two patients with acute necrotizing encephalopathy without hemorrhage, the lesions appeared as bilateral thalamic, bilateral occipital subcortical, bilateral temporal cortical and subcortical T2 and FLAIR hyperintense SI.Bilateral thalami damage is often a distinctive feature in ANE [21]. Hemorrhage in thalamic lesions is an additional MRI features suggestive of ANE, but may be missing, without ruling out the diagnosis [22].

Wong et al. [22] observed that the absence of hemorrhage in ANE seems to be with better outcome, while in our study the included patients died (*p* value 0.506).

Respiratory virus-induced neuro-immunopathology due to a dysregulation of host immune response has been described, in particular for ANE; this can be induced by the cytokinic storm secondary to viral infections [23].

Neurotoxicity triggered by immune response to COVID-19, associated with IgG targeting a neuronal antigen found in fiber tracts, is suspected here. These antibodies may invade an autoantigen through molecular mimic to the virus. Destruction of the cells produces releasing large amounts of autoantigens; also this may stimulate self-reactive cells and lead to self-reactive antibodies. After COVID-19 infection, the inflammatory storm could contribute the production of IgG and interrupt the blood–brain barrier, thus causing an ANE [22].

The study had its limitations, primarily, as with all retrospective studies; it was subject to systematic review confounders. MR examinations were exclusively conducted in critically ill patients, hence the natural existence of indication bias.

The lack of control group undermined the specificity of findings to COVID-19 and limited our drawn conclusions. The lack of histologic and pathologic assessment was also considered one of our main study limitations.

Some of our patients with mild neurological symptoms didn’t undergo to imaging due to regulatory constraints imposed during the COVID-19 pandemic, and on the contrary, other patients with more obvious neurologic impairment may have been too unstable to undergo imaging.

Finally, our study was not comprehensive to a few potential areas of interest. For example, intracranial vasculitis could be a rational finding of infection, but, due to absence of MR angiography with contrast in our cohort, we were unable to assess the prevalence of intracranial vasculitis.

## Conclusions

Neurological complications were significantly detectable in the total observed COVID-19 patients during the designed study time frame.

The frequentness of acute/subacute stroke was ordered first and then parenchymal hematomas and hemorrhage were second findings. Other diagnoses in order of frequentness include PRES, WM leuko-encephalopathic involvement and microhemorrhage and then cerebral edema, vascular thrombosis and acute necrotizing encephalitis.

In general, an outstanding bad prognostic outcome was prevailed in COVID-19 patients associated with neurological manifestations.

## Data Availability

The data sets used and/or analyzed during the current study are available from the corresponding author on reasonable request.

## Abbreviations

COVID-19: Coronavirus disease 19
SARA-COV2: Severe acute respiratory syndrome coronavirus
ARDS: Acute respiratory distress syndrome
CNS: Central nervous system
rRT-PCR: Real-time reverse transcription polymerase chain reaction
CT: Computed tomography
MRI: Magnetic resonance image
3D T1WI FSE: Three-dimensional T1-weighted image fast spin echo
FLAIR: Fluid-attenuated inversion recovery
DWI: Diffusion-weighted imaging
ADC: Apparent diffusion coefficient
SWI: Susceptibility-weighted imaging
CABG: Coronary artery bypass graft
SAH: Subarachnoid hemorrhage
PRES: Posterior reversible encephalopathy syndrome
WM: White matter
CSF: Cerebrospinal fluid
ACE2: Angiotensin-converting enzyme
ANE: Acute necrotizing encephalopathy
